# Neuroinflammation and Copper in Alzheimer's Disease

**DOI:** 10.1155/2013/145345

**Published:** 2013-11-28

**Authors:** Xin Yi Choo, Lobna Alukaidey, Anthony R. White, Alexandra Grubman

**Affiliations:** Department of Pathology, University of Melbourne, VIC 3010, Australia

## Abstract

Inflammation is the innate immune response to infection or tissue damage. Initiation of proinflammatory cascades in the central nervous system (CNS) occurs through recognition of danger associated molecular patterns by cognate immune receptors expressed on inflammatory cells and leads to rapid responses to remove the danger stimulus. The presence of activated microglia and astrocytes in the vicinity of amyloid plaques in the brains of Alzheimer's disease (AD) patients and mouse models implicates inflammation as a contributor to AD pathogenesis. Activated microglia play a critical role in amyloid clearance, but chronic deregulation of CNS inflammatory pathways results in secretion of neurotoxic mediators that ultimately contribute to neurodegeneration in AD. Copper (Cu) homeostasis is profoundly affected in AD, and accumulated extracellular Cu drives A**β** aggregation, while intracellular Cu deficiency limits bioavailable Cu required for CNS functions. This review presents an overview of inflammatory events that occur in AD in response to A**β** and highlights recent advances on the role of Cu in modulation of beneficial and detrimental inflammatory responses in AD.

## 1. Inflammation

Inflammation is a protective response rapidly triggered by innate immune cells in the event of tissue injury, as well as endogenous or exogenous insults (reviewed in [1]). The process is highly complicated, involving the complex interplay of cells and mediators. In brief, acute inflammatory responses involve vasodilation to increase blood flow combined with alterations in microvascular structure to allow exit of circulating leukocytes and plasma proteins, followed by accumulation and activation of leukocytes at the site of injury, where leukocyte extravasation is largely facilitated by cytokines including tumour necrosis factor (TNF) and interleukin-1 (IL-1) [1]. In addition, activated innate immune cells at site of injury remove cellular debris and/or pathogens via phagocytosis with concomitant cytokine production to facilitate the initiation of adaptive responses [1].

Due to the variability in the nature, severity, and site of injuries, resolution of inflammatory processes, where all injury and insults become resolved with little tissue damage, is not always possible. For severe tissue damage where regeneration is insufficient, healing with fibrosis may occur instead. The third possible outcome is progression from acute to chronic inflammation. This occurs when danger signals persist and inflammation cannot be resolved. Notably, a wide range of diseases, including asthma [2], diabetes [3], coronary heart disease [4], cancer [5], and neurodegenerative diseases [6, 7], have been associated with chronic inflammation.

### 1.1. Inflammatory Signaling Cascades

The innate immune system functions as the first line of defense against cellular damage caused by danger stimuli including pathogenic organisms or damaging molecules. An array of innate immune cells including macrophages, mast cells, fibroblast, dendritic cells, monocytes, and neutrophils are involved in inflammatory responses. Innate immune cells sense danger signals by activation of membrane-bound pattern recognition receptors (PRRs), including Toll-like receptors (TLRs) and C-type lectin receptors (CLRs), and cytoplasmic PRRs, including nucleotide-binding oligomerization domain (NOD)-like receptors (NLRs) and RIG-I-like receptors (RLRs), to initiate immune responses [8]. Activation of PRRs by pathogen-associated molecular patterns (PAMPs), usually conserved molecular patterns expressed by pathogens, and/or danger-associated molecular patterns (DAMPs), endogenous molecules released by damaged cells, triggers inflammatory signaling cascade(s) that drive a wide range of cellular responses [1].

The TLR family, with 10 identified members, is the most widely studied of all classes of PRRs [9]. TLRs are type 1 transmembrane proteins with an extracellular domain with leucine-rich repeats (LRRs) and a cytoplasmic Toll/IL-1 receptor domain [10]. TLRs play an important role in detecting microbial infection via recognition of ligands including lipids, nucleic acids, lipopolysaccharides, and other unique molecular microbial components [11, 12]. TLRs undergo conformational changes upon ligand binding to recruit adaptor molecules, which in different combinations contribute to the specificity in individual TLR responses [13]. The two major signaling pathways initiated by TLRs include the myeloid differentiation primary response gene 88 (MYD88)-dependent pathway and the Toll/interleukin-1 receptor (TIR)-domain-containing adapter-inducing interferon-*β* (TRIF)-dependent pathway [1]. MYD88-dependent signaling responses activate the c-Jun N-terminal kinases (JNK)/activator protein 1 (AP-1) and kappa-light-chain-enhancer of activated B cells (NF-*κ*B) pathways [9]. AP-1 and NF-*κ*B activation initiates a transcriptional response program of cytokine production specifically tailored to the insult. Classical NF-*κ*B-regulated cytokines include IL-1, IL-6, IL-8, and TNF [14]. In the alternative TRIF-dependent pathway, activation of the transcription factors NF-*κ*B and interferon regulatory factors (IRF3 and IRF7) can result in type 1 interferon (IFN) production, often in response to viral infection [1]. Type I IFNs are released by various peripheral cell types, including lymphocytes such as natural killer (NK) cells, B cells and T cells, macrophages, fibroblasts, and endothelial cells, and are likely to induce CNS inflammatory effects through infiltration of peripheral cells into the brain [15].

In contrast to the TLRs, NLRs are cytosolic PRRs that can be activated by both pathogens and endogenous components [16]. Making up the NLR family are 5 NOD receptors, 14 NALP receptors, NAIP, Ipaf, and CIITA type 1 and CIITA receptors [17]. In particular, NOD1 and NOD2, best known for their intercellular sensing of distinct peptidoglycan fragments released by bacteria, also signal to NF-*κ*B [18] and mitogen-activated protein kinase (MAPK) [19] signaling pathways. On the other hand, NLRP1, NLRP3, and NLRC4 recognise specific PAMPS and DAMPS to form signaling complexes known as “inflammasomes.” Inflammasome assembly may be triggered by diverse stimuli, including uric acid crystals, cholesterol, protein aggregates and, aluminium adjuvants [20–23]. Assembly of “inflammasome” complexes leads to caspase-1 activation resulting in caspase-1-mediated cleavage of NF-*κ*B-dependent precursors of the proinflammatory cytokines IL-18 and IL-1*β* to produce their mature forms [1, 19]. Therefore inflammasome activation requires two signals, the first to induce transcription of pro-IL-1*β* and pro-IL-18 and the second to initiate inflammasome assembly.

Overall, inflammatory responses can be viewed as a system consisting of mediator-driven feedback loops. Subsets of inflammatory mediators can positively feedback into the system to intensify activation state of immune cells, leading to exacerbated inflammatory responses [24, 25]. Conversely, other inflammatory mediator subsets can function in a reverse manner. They can negatively feedback into the system leading to inhibited or downregulated inflammatory responses so to limit tissue injury for the resolution of inflammation [26]. Depending on the mediator secretion profile, inflammation can mediate different outcomes. From the brief outline of the inflammatory signaling cascades, it is evident that inflammation is a highly complex but tightly regulated process. More importantly, the regulation of inflammation can vary in different organs, due to tissue-specific expression of innate receptors and variations in inflammatory mediator secretion profiles.

## 2. Neuroinflammation

Being physically separated from the peripheral immune system, the CNS is conventionally recognized as being “immunologically privileged” [34]. With limited regenerating capacity, tight regulation of the immune responses in the CNS is necessary as chronic inflammatory responses in the CNS can lead to sustained neurodegeneration [35]. Immune regulation of the CNS is characterised by the absence of defined lymphatic channels, downregulated immune surveillance, absence of specialized antigen presenting cells, and the presence of the blood-brain barrier (BBB) [34, 36, 37]. Aside from its functions as a physical barrier to separate cerebrospinal fluid from circulating blood, the BBB also serves as a specialized physical barrier to limit immune responses.

Despite the immunologically privileged status, it is becoming increasingly recognized that the CNS is capable of independently shaping immune responses [37]. Lymphocytes, in particular T cells, can be trafficked into the CNS to survey the environment [37, 38]. Emerging evidence suggests that a lymph-like system is also present in the brain [36, 39]. But most importantly, specialized cells of the CNS express major histocompatibility complexes (MHC) classes I and II molecules and can be activated to participate in immune responses [37].

In an analogous manner to systemic organs, inflammatory reactions in the CNS play a critical role in maintaining tissue homeostasis [40]. Neuroinflammatory responses can also be classified as acute or chronic. Acute neuroinflammation usually occurs in reversible neuronal injury where glial cells in the CNS become activated [41]. The process is usually short-lived and responses by glial cells are generally subtle. On the other hand, chronic inflammation refers to prolonged inflammatory responses in the CNS due to a persistent presence of injurious stimuli. Notably, neuroinflammation described in diseases of the CNS, including neurodegenerative diseases, is generally chronic [41]. Despite differences in their clinical presentation and underlying mechanisms of disease, neuroinflammation has been identified as a process crucial to the progression of many neurodegenerative diseases including AD, motor neuron disease (MND), and Parkinson's disease (PD). This is supported by the observation in models of neurodegenerative disease, as well as patient tissues, of activation and/or proliferation of glia, the major cell types initiating the neuroinflammatory process [42–48].

In conclusion, the understanding of neuroinflammatory processes occurring in neurodegenerative diseases raises many as yet unanswered questions: does the inflammatory process occur before or after the occurrence of other pathological changes? Are the inflammatory processes beneficial or detrimental to disease progression? Nevertheless, it is clear that if neuroinflammation remains unresolved, as is the case for most neurodegenerative diseases, prolonged exposure to cytokine-induced injury is neurotoxic. As the clinical and pathological presentation of various neurodegenerative diseases can be very different, there are also disease-specific differences in the associated inflammatory profiles. Therefore, further understanding the triggers and consequences of inflammatory processes in a disease-specific context will have strong implications for potential therapeutic approaches.

### 2.1. Neuroinflammation in AD

**Figure 1:**
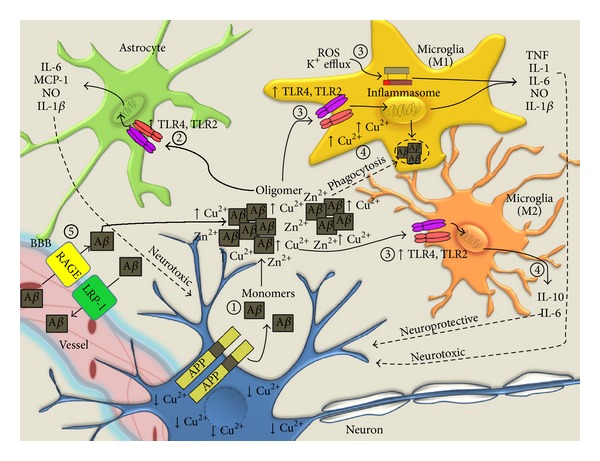
*Inflammatory processes in AD*. (1) Sequential cleavage of APP by secretase proteins generates extracellular A*β* monomers, which aggregate to form toxic oligomers and plaques, a process accelerated by Cu and Zn ions. (2) A*β* oligomers may directly interact with immune components on astrocytes, potentially through TLR2/4 recognition, resulting in astrocyte-derived secretion of toxic proinflammatory mediators that act on neurons. (3) Microglia surrounding A*β* plaques may be polarised to the neurotoxic M1 phenotype through A*β*- or ROS-dependent inflammasome and TLR activation. (4) Microglia may also exert protective functions through intracellular Cu sequestration, direct phagocytic activity on plaques, and secretion on neuroprotective M2 mediators including IL-10. (5) The brain levels of A*β* are also controlled by RAGE- and LRP-1 mediated transport between the plasma and brain. Increased vascular RAGE in AD contributes to impaired clearance of A*β* from the CNS.

There was little evidence to link neuroinflammation to disease progression of AD until immunohistochemistry techniques were used to study amyloid *β* (A*β*) plaques. Towards the end of the 1980s, several research groups detected clusters of activated microglia around A*β* plaques [49–51]. These observations support the hypothesis that A*β* plaques are key players in chronic neuroinflammation in AD. Later, it was suggested that the presence in AD brains of high levels of redox metals, including iron (Fe) and copper (Cu), promotes reactive oxygen species (ROS) production and these metals can also act as mediators to induce neuroinflammation in AD [52, 53]. Specifically, Cu concentrations are elevated in amyloid plaques and Cu binds with A*β* high affinity, thereby promoting A*β* oligomerization and neurotoxicity (Figure 1).

Further studies on the neuroinflammatory profile in AD also revealed abnormal upregulation of various cytokines and chemokines including TNF, IL-1, IL-6, monocyte chemoattractant protein-1 (MCP-1), nitric oxide (NO), and transforming growth factor *β* (TGF*β*), which exert proinflammatory effects in AD brain [54–57]. Put together, these data suggest that neuroinflammation has a significant impact on the disease pathology of AD (for detailed reviews, see [58, 59]).

Ageing is still considered the greatest risk factor for AD although risk modifiers including environmental, genetic, and epigenetic factors have been described. Among the physiological and lifestyle changes during ageing, alterations to metal homeostasis and inflammatory processes have also been documented, which may have a causal connection with increased AD risk (reviewed in [60]). Plasma concentrations of copper are elevated in ageing [61], which may contribute to metal imbalances that trigger ROS production and A*β* aggregation. Moreover, inadequate zinc absorption during ageing contributes to low-grade peripheral inflammation due to deregulated inflammatory transcription factors (including NF-*κ*B) containing zinc-finger motifs [60]. Thus the compounding effects of metal and inflammatory dyshomeostasis in ageing may impede normal physiological responses to additional stressors such as increased A*β* deposition.

### 2.2. Cell Types Involved

#### 2.2.1. Microglia

The key innate immune cell types mediating inflammatory process of AD brain include microglia and astrocytes. Microglia are the major immune surveillance cells accounting for 10–20% of the total CNS glial cell population. Also known as resident macrophages, microglia are of mesodermal origin and known to occupy all regions of the CNS, although microglial density can vary widely in different regions of the CNS [62, 63]. Microglial activity can vary between populations and regions of the CNS, based on differences in gene expression profiles [64]. Additionally, microglia also express different phenotypes depending on the surrounding environmental conditions. In healthy individuals, microglia have a “resting” phenotype, characterised by a ramified morphology, which are in constant motion, surveying their microenvironment [64]. In AD, microglia migrate to areas of A*β* plaque deposition [49–51] and were shown to participate in the clearance of A*β* plaques via phagocytosis or A*β* plaque degradation [65, 66]. While clearance of A*β* by microglia is suggestive of a beneficial response, the actual outcome of A*β* clearance by microglia remains unknown. Rather, phagocytosis of A*β* by microglia may drive further immune activation, as supported by increased microglial production of proinflammatory mediators, including IL-1 [57, 67, 68], IL-6 [57], TNF [57], macrophage inflammatory protein (MIP) [57], MCP-1 [57, 67], and ROS [69]. However, the precise role of microglia in the pathogenesis of AD has yet to be elucidated. This is due, in part, to the multiple activation states that can be expressed by microglia. Similarly to peripheral macrophages, microglia can be classically (M1) or alternatively (M2) activated. In brief, M1 microglia are primarily associated with proinflammatory responses and induce neurodegeneration, while M2 phenotypes are more closely associated with anti-inflammatory responses, which are neuroprotective [70]. In an *in vivo* study involving the APP/PS1 AD mouse model, an age-dependent switch of microglial activation state, from M2 to M1, was observed [71]. However, this does not rule out that both M1 and M2 populations can coexist and play varying roles in AD disease pathogenesis (Figure 1).

#### 2.2.2. Astrocytes

Astrocytes, of ectodermal origin, are the most abundant glial cell type and the most abundant cell type in the CNS. They can be classified by morphological differences into protoplasmic and fibrous astrocytes, which are present in the gray and white matter regions, respectively [72]. It is well established that astrocytes play critical roles in supporting neuronal survival and maintaining homeostasis of the CNS by close contact with all CNS cell types through their extensive processes. Emerging evidence suggests that astrocytes also play a role in the innate immune system [73]. In the presence of injurious stimuli, astrocytes can be triggered to initiate immune responses. Common observation of astrocyte clusters around A*β* plaques in AD brains suggests that A*β* plaques are key endogenous stimuli driving reactive astrocytosis [74]. This is further supported by *in vitro* evidence of astrocyte activation in response to aggregated A*β* fragments [75]. In contrast to microglia, astrocytes do not play a significant role in the clearance of A*β* plaques [65, 66]. Instead, astrocytes downregulate microglial A*β* plaque clearance via secretion of glycosaminoglycans-sensitive molecules [65, 66]. This implies that astrocytes may act to hinder effective clearance of A*β*, indirectly promoting A*β* accumulation in AD brains. However, a recent study carried out in adult astrocytes suggests that astrocytes can contribute to A*β* degradation [76]. Additionally, astrocytes play significant role in AD neuroinflammation by producing a broad range of inflammatory mediators, including IL-1*β* [77, 78], IL-6 [79], MCP-1 [78], MIP [78], and NO [80], identified from various *in vivo* and* in vitro* studies. Long-term production of these mediators becomes a chronic “cytokine cycle” (Figure 1), as described by Griffin and colleagues, which plays detrimental role in influencing disease progression [24]. Therefore cytokine-induced feedback loops may present a target for therapeutic intervention using anti-inflammatory approaches.

### 2.3. Pathways and Mediators

Many studies report that A*β* oligomers and fibrils are key drivers of AD pathogenesis [81, 82]. Besides causing direct injury to neuronal cells [81, 83], A*β* oligomers and fibrils are endogenous stimuli that can be recognized by PRRs expressed on innate immune cells. A*β* species have been shown to induce inflammatory responses through activating various PRRs expressed by microglia and astrocytes including TLRs [84, 85], receptor for advanced glycation end products (RAGE) [86, 87], and the inflammasomes [23, 88].

#### 2.3.1. TLR Signaling

Microglia and astrocytes can be differentiated based on the TLRs they express. Microglia express all TLRs 1–9, while astrocytes predominantly express TLR3, although low-level expression of TLR1, TLR4, TLR5, and TLR9 has also been detected [89, 90]. In particular, TLR2 [85] and TLR4 [84, 91] have been identified to be important for the recognition of A*β* species in AD. TLR activation by A*β* can function as a double-edged sword. Activation of TLR2 and TLR4 was shown to be beneficial through enhanced phagocytic microglial A*β* clearance, and TLR2 or TLR4 deficiency in AD mice has detrimental effects on A*β* deposition and cognitive function [92, 93]. However, TLR2 knockout and TLR4 loss-of-function mutant mouse models secreted less neurotoxic proinflammatory mediators IL-1*β*, IL-6, TNF, and inducible nitric oxide synthase (iNOS) with A*β* stimulation, suggesting that TLR-dependent signaling may contribute to neurotoxicity in AD [84, 85]. This is supported by further evidence showing that A*β* can initiate sterile inflammation via heterodimeric TLR4/TLR6 when accompanied by regulatory signals from scavenger receptor CD36 [94]. Additionally, brains of human AD patients express high levels of TLR4, while APP mouse brains exhibit higher levels of *TLR4* mRNA [84]. Recently, upregulated TLR2 and TLR4 have been detected in peripheral mononuclear blood cells in 60 patients with late onset AD [95]. These studies suggest that inflammatory responses in AD brains can be further potentiated by A*β*-induced upregulation of TLR4 expression. Although TLRs can activate several transcription factors, including AP-1 and NF-*κ*B [1, 9], the current understanding of the downstream signaling cascades in AD is limited.

#### 2.3.2. RAGE Signaling

RAGE, a member of the immunoglobulin superfamily of cell surface proteins, is a multiligand receptor that functions as a PRR for A*β* oligomers [96]. RAGE exists in a membrane-bound full-length form and a soluble form (sRAGE) that competitively inhibits A*β*-mediated RAGE signaling. Emerging evidence suggests the involvement of RAGE in AD pathogenesis. RAGE promotes A*β* transport from plasma to the CNS (Figure 1). Conversely, low-density lipoprotein receptor related protein 1 (LRP-1) exerts the reverse function and increases plasma A*β* levels [97]. Thus the combined actions of RAGE and LRP-1 maintain the balance of plasma and CNS A*β* concentrations [97]. Significant elevation of the hippocampal microvascular ratio of RAGE to LRP-1 expression was reported in AD patients [98], resulting in impaired clearance of A*β* in AD with RAGE driving the influx of A*β* into the CNS [97]. Moreover, reduced sRAGE expression in AD further contributes to overactive RAGE-induced inflammation. Additionally, studies also revealed that A*β* binding to RAGE drives microglial activation, thereby initiating a positive feedback loop that further elevates RAGE expression and associated inflammation [87]. APP mutant AD model mice crossed with mice overexpressing RAGE demonstrated exacerbated disease outcomes, indicating that elevated RAGE expression is detrimental to cognitive function in AD [86, 99].

#### 2.3.3. NLR Signaling

Another class of PRR involved in AD pathogenesis is the NLRs. In particular, IL-1*β* and IL-18, predominant cytokines released with inflammasome assembly, are significantly upregulated in both CNS and plasma components of human AD patients [100, 101]. In addition, IL-1*β* was also found to be significantly upregulated in the Tg2567 mouse model of AD [102]. These data support the involvement of inflammasome activation in AD neuroinflammation. NALP3 inflammasome activation can be induced by potassium (K^+^) efflux [103]. In particular, A*β* species have been shown to induce reduced intracellular K^+^ by disrupting K^+^ channel function [104, 105]. Treatment of hippocampal neurons with A*β* induced upregulation of the KV3.4 channel subunit and increased K^+^ efflux, and this is also evident in Tg2576 mouse model. In addition to the above, the NALP3 inflammasome can also be directly activated by A*β* upon phagocytosis of A*β* by microglia [23] due to consequential triggering of lysosomal damage [106].

Despite the rapidly increasing knowledge of the inflammatory cells, pathways, and mediators significantly altered in AD, several major questions remain unanswered. Critical to our understanding of the disease process as well as development of diagnostic and therapeutic tools is a clearer picture of the specific triggers of microglial activation in AD and the pathways that can be induced to shift microglial responses to protective M2 phenotypes. Moreover, a spatiotemporal analysis of the beneficial and detrimental consequences of microglial activation in AD is required to target pathways that selectively engage protective responses such as phagocytosis of amyloid deposits while limiting secretion of neurotoxic mediators.

## 3. A Role for Cu in Neuroinflammation in AD

### 3.1. Cu and A*β* in AD

**Figure 2:**
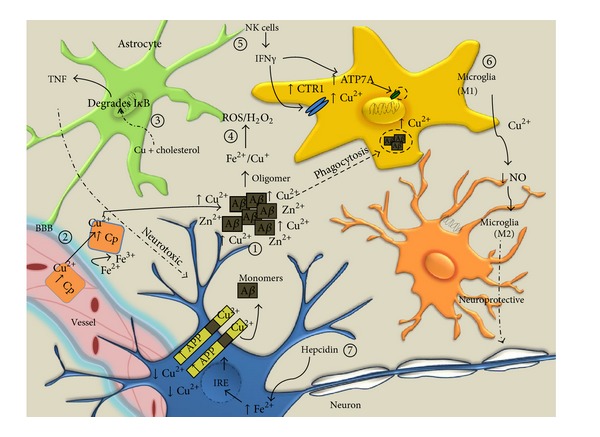
*Hypothesised roles of copper in the inflammatory process of AD.* (1) Clockwise from center. Cu and Zn induce the aggregation of A*β* in AD, leading to reduced neuronal intracellular bioavailable Cu. This may account for the reported reduced expression of the Cu-requiring proteins SOD1 and ATOX1 in AD. (2) Cp, the Cu-transport protein, which is elevated in AD, can promote Fe oxidation, inflammation [28], and increased extracellular Cu levels in the CNS. (3) Copper has been shown to potentiate the effects of cholesterol on inflammation-induced A*β* neurotoxicity through increased TNF production [30]. (4) APP or A*β* reduce Cu^2+^ to Cu^+^—this redox cycling promotes production of ROS including H_2_O_2_. (5) NK cell-derived IFN*γ* can increase Cu uptake in microglia via enhanced CTR1 expression [27]. IFN*γ* also promotes ATP7A elevation and vesicular trafficking. These mechanisms of Cu sequestration by microglia may prevent further plaque formation. Phagocytosis of amyloid plaques also raises microglial Cu levels and promotes A*β* clearance. (6) Cu may polarize inflamed microglial populations from the neurotoxic (M1) phenotype to the neuroprotective (M2) phenotype via inhibition of NO production [31]. (7) The Fe master regulator, hepcidin, is induced by cytokines in AD and prevents Fe release from neurons. Excess Fe binds IREs in the APP promoter and upregulates APP production, promoting Cu export and mislocalisation.

Cu is essential for the development and maintenance of CNS functioning. It is becoming well established that deregulation of Cu homeostasis is a pathological feature associated with a number of neurodegenerative diseases including AD, PD, and MND [107]. Although the precise role(s) that Cu plays in the pathology of these diseases is not elucidated, Cu is a critical cofactor of numerous enzymes but in excess can mediate Fenton chemistry-dependent cytotoxicity and therefore must be tightly regulated [108]. Cu plays an important role in AD pathology by a twofold mechanism involving toxic Cu-induced A*β* deposition occurring concomitantly with reduced intracellular bioavailable Cu [32, 109–111] (Figure 2). Additionally, Zn is also reported to potently induce A*β* plaque deposition [112] and Fe can mediate ROS production [113]; thus it is not surprising that changes to homeostasis of both Zn and Fe in AD have been described [110]. As discussed above, although the mechanisms by which A*β* exerts its toxic effects on neurons are not fully elucidated, ROS and acute inflammatory mediators produced by glial cells enriched at amyloid plaques may contribute to A*β*-induced neuronal death.

Several *in vitro* studies have demonstrated that low levels of Cu ion can induce A*β* aggregation [114–117]. Cu can bind with high affinity to an amino terminal tyrosine residue in A*β* and induce oligomerization through oxidative modification [118]. APP knockout mice exhibit elevated brain Cu levels, whereas APP overexpressing transgenic mice have reduced brain Cu levels [32, 119]. APP or A*β* interactions with Cu^2+^ induce reduction to Cu^+^ *in vitro*, promoting neurotoxic H_2_O_2_ production [120]. It is now well established that Cu binding induces A*β* deposition and promotes neurotoxicity [118]. Additionally, elevated free Cu can mediate ROS-dependent toxicity [53]. As chronic activation of inflammatory cells is commonly observed in the vicinity of A*β* plaques [49–51], it stands to reason that an agent that drives A*β* deposition would indirectly contribute to damaging chronic immune responses. However, as there is controversy regarding the role of activated microglia surrounding amyloid plaques, it is unclear whether the contribution of Cu to this process is beneficial.

### 3.2. Proinflammatory Role of Cu

A role for Cu in peripheral inflammatory responses is supported by *in vivo* and *in vitro* data. Cu was reported to induce IL-6 secretion in a cell culture system composed of human keratinocytes and fibroblasts [121]. Implantation of female rats with Cu-coated discs caused NF-*κ*B activation and IL-6 production and induced recruitment of IL-1*α*-secreting cells [122, 123]. Inhalation of Cu present as a particulate in air pollution also elicited inflammatory NF-*κ*B activation [124]. Moreover, intratracheal instillation of Cu sulfate results in enhanced neutrophilia and MIP2 mRNA expression in rats [125] and Cu chloride can elicit IL-8 responses in human endothelial cells [126]. Together, these studies demonstrate that Cu can induce peripheral inflammation in numerous models, although there is limited direct evidence of the potential of Cu to initiate neuroinflammation. Synergistic effects of Cu and cholesterol in neuroinflammation have been described. A study demonstrated that trace Cu potentiated A*β* neurotoxicity in cholesterol-fed mice by A*β*-induced neurotoxic inflammatory responses [30] (Figure 2). No proinflammatory effects were observed upon treatment with Cu or cholesterol alone. However, cotreatment with Cu and cholesterol increased I*κ*B degradation as well as TNF expression and production in the brains of the mice, implicating a TNF-dependent proinflammatory role for Cu and cholesterol in AD. Investigation of the role of cholesterol in Cu-induced inflammatory responses would provide critical insight into AD pathogenesis, as genetic variation in the cholesterol transport protein ApoE4 remains the greatest genetic risk factor for sporadic AD and cholesterol-rich lipid rafts are likely to be the site of Cu-A*β* interactions (reviewed in [127]). Moreover, AD-associated changes to cholesterol metabolism may impact membrane fluidics, which could affect associated copper transporter and PRR expression, as well as inflammatory signal transduction pathways.

### 3.3. Cu Trafficking and Inflammation

The major Cu-binding proteins in plasma are ceruloplasmin (Cp), albumin, and transcuprein, while subcellular Cu transport is controlled by membrane transporters and cytosolic chaperones that shuttle Cu between intracellular compartments and high-affinity cuproproteins, respectively. Loss of the Cu transporter ATP7B in Wilson's disease model mice presents as elevated brain copper, neurodegeneration, and inflammation [128]. Interestingly, ATP7B gene polymorphisms have been associated with increased AD risk in certain populations [129, 130]. Moreover, an inflammatory milieu can affect Cu homeostasis via regulation of Cu transport proteins (Figure 2). IFN*γ*, which is secreted by NK cells in AD patients [131], stimulated ATP7A expression in cultured microglia and altered Cu homeostasis, including Cu-dependent trafficking of ATP7A from the Golgi to cytoplasmic vesicles [27]. IFN*γ* stimulation also increased Cu uptake and elevated expression of the CTR1 Cu importer [27]. The impact of this finding for AD is unclear, as IFN*γ* exerts multiple biological effects in AD. Aside from stimulating microglial activation, IFN*γ* was also reported to mediate neurogenesis and reduce Tau pathology in AD model mice [132, 133]. Zheng et al. [27] also reported elevated ATP7A expression in activated microglia surrounding A*β* plaques in the brains of TgCRND8 AD model mice [27], which may promote overall Cu uptake by upregulation of CTR1 expression. Cu sequestration by microglia may therefore provide a neuroprotective mechanism in AD by limiting the free extracellular Cu available to seed A*β* aggregation and plaque formation. Conversely, transfection of fibroblast cell lines with ATP7A resulted in loss of cellular copper and reduced APP expression [33], suggesting that it is the lack of intracellular copper induced by elevated ATP7A that may prevent A*β* production by downregulation of APP.

The major plasma Cu transport protein, Cp, which is elevated in the serum and brain in AD patients [134, 135], can also elicit proinflammatory responses in cultured primary and secondary microglia. These inflammatory responses include elevated NO release and induction of proinflammatory transcriptional programs involving TNF, IL-1*β*, COX-2, NADPH oxidase, iNOS, and prostaglandin E2 [28]. Cu-stimulated responses were significantly attenuated by a p38 inhibitor, SB203580, and the NF-*κ*B inhibitor SN50 [28]. Moreover, as Cp is a Cu-containing ferroxidase [136], transport of Fe is inextricably linked to Cu mislocalisation and Cp levels.

The Fe regulating peptide, hepcidin, which is induced by cytokines including IL-6, inhibits Fe release from neurons by inducing lysosomal degradation of the Fe exporter, ferroportin [137]. A recent study proposed that the resultant intracellular excess Fe in ageing, which is further exacerbated by inflammation in AD, promotes APP production via an iron responsive element (IRE) in the promoter of APP [138, 139]. Enhanced APP expression thereby promotes neuronal Cu export, as APP binds Cu via its Cu-binding domain [139]. Cu mislocalisation by secreted APP could therefore act as a double-edged sword resulting in an excess extracellular Cu that may promote A*β* aggregation and deplete intracellular Cu stores available for physiological enzyme functions. However, another study reported that Cu but not Fe induced APP exocytosis *in vitro* [140], suggesting that complex regulatory mechanisms, which may be dependent on the surrounding inflammatory milieu, may be important for subtle control of APP trafficking.

### 3.4. Anti-Inflammatory Role of Cu in AD

Conversely, intracellular Cu deficiency, which was detected in brain of mice overexpressing APP [32, 109] and supported by studies of AD brain tissue [111], also promotes microglial activation. A loss of bioavailable brain Cu, in mice that had been perinatally weaned on Cu deficient diets, resulted in microglial and astrocytic activation in the cerebrum and thalamus and neurological signs [141]. This suggests that bioavailable physiological Cu concentrations are required to prevent CNS inflammation. For instance, expression of the Cu-requiring enzymes superoxide dismutase 1 (SOD1) and the ATX antioxidant protein homolog (ATOX1) was significantly reduced in AD brains as determined by several microarray studies, suggesting that neurons are Cu-deficient in AD [139, 142, 143]. As SOD1 itself may also exert anti-inflammatory functions through ROS detoxification, a loss of SOD1 activity would further exacerbate chronic inflammation.

Cu is also closely associated with regulation of cytokine signaling. Robust secretion of the anti-inflammatory cytokine, IL-4 was detected in the brains of mice coadministered Al and Cu in drinking water compared to mice administered Al alone [29]. Interestingly Cu administration alone had no effect on the inflammatory markers tested, although the study did not examine whether Cu was increased in the brain as a result of treatment. Stimulation of the microglial BV2 cell line with LPS in the presence of Cu(I) shifted the population from the neurotoxic M1 to the neurotrophic M2 phenotype and significantly reduced nitrite release [31]. Treatment with Cu alone (without LPS) had no effect on the microglial phenotype, nitrite release, or iNOS expression, thereby suggesting that Cu(I) can modulate inflamed microglia and may alter the cell signaling function of NO by altering its redox state. NO is an M1 mediator, and Cu-dependent inhibition of nitrite release may be the mechanism, which induces the M2 phenotype. Therefore, Cu sequestration by microglia may be a neuroprotective response in activated microglia surrounding A*β* plaques. These neuroprotective responses may be mediated, at least in part, by Cu-dependent induction of the metal-sequestering and antioxidant acute phase protein, metallothionein, as observed in rat microglia [144]. Administration of the therapeutic copper-bis(thiosemicarbazonato) complex, Cu(gtsm), delivered bioavailable Cu to the brain of APP/PS1 AD model mice and improved amyloid and tau pathology, as well as indicators of cognitive function [145]. As Cu(gtsm) is able to cross the BBB and release Cu inside brain cells, this study further supports the hypothesis that intracellular Cu pools are depleted in AD. It will be interesting to determine whether Cu delivery is anti-inflammatory or promotes M1 to M2 shift in the activated microglia surrounding amyloid deposits in these mice as part of the neuroprotective mechanism.

## 4. Conclusions

As discussed above, extensive evidence links neuroinflammation to AD. Whilst regulated neuroinflammation is an important neuroprotective mechanism in the CNS, unregulated, chronic neuroinflammation is toxic if not resolved as in AD. Cu appears to possess both pro- and anti-inflammatory properties that may be mediated in part by its spatial proximity to amyloid plaques. Thus deregulation of Cu transport may be the precursor to initiation of toxic inflammatory reactions in the AD brain. Future investigation of the effects of both elevated and depleted discrete Cu pools on inflammatory pathways, as well as improved techniques for measurement of subcellular Cu trafficking in AD, will facilitate identification of novel therapeutic targets related to Cu homeostasis and inflammation.

## Abbreviations

A*β*: Amyloid *β*
AD: Alzheimer's disease
AP-1: Activator protein 1
BBB: Blood-brain barrier
CLRs: C-type lectin receptors
CNS: Central nervous system
Cp: Ceruloplasmin
Cu: Copper
DAMPs: Danger-associated molecular patterns
Fe: Iron
IFN: Interferon
IL-1: Interleukin-1
iNOS: Inducible nitric oxide synthase
IRF: IFN regulatory factor
JNK: c-Jun N-terminal kinases
LRP1: Lipoprotein receptor related protein 1
LRRs: Leucine-rich repeats
MAPK: Mitogen-activated protein kinase
MCP-1: Monocyte chemoattractant protein-1
MHC: Major histocompatibility complexes
MIP: Macrophage inflammatory protein
MND: Motor neuron disease
MYD88: Myeloid differentiation primary response gene 88
NK: Natural killer
NF-*κ*B: Kappa-light-chain-enhancer of activated B cells
NLRs: NOD-like receptors
NO: Nitric oxide
NOD: Nucleotide-binding oligomerization domain
PAMPs: Pathogen-associated molecular patterns
PD: Parkinson's disease
PRRs: Pattern recognition receptors
RLRs: RIG-I-like receptors
RAGE: Receptor for advanced glycation end products
ROS: Reactive oxygen species
sRAGE: Soluble RAGE
TGF*β*: Transforming growth factor *β*
TIR: Toll/interleukin-1 receptor
TLRs: Toll-like receptors
TNF: Tumour necrosis factor
TRIF: TIR domain-containing adapter-inducing interferon-*β*
Zn: Zinc.

## References

[1] Newton K, Dixit VM (2012). Signaling in innate immunity and inflammation. *Cold Spring Harbor Perspectives in Biology*.

[2] Murdoch JR, Lloyd CM (2010). Chronic inflammation and asthma. *Mutation Research*.

[3] Xu H, Barnes GT, Yang Q (2003). Chronic inflammation in fat plays a crucial role in the development of obesity-related insulin resistance. *Journal of Clinical Investigation*.

[4] Danesh J, Whincup P, Walker M (2000). Low grade inflammation and coronary heart disease: prospective study and updated meta-analyses. *British Medical Journal*.

[5] Fillon M (2013). Details linking chronic inflammation and cancer continue to emerge. *Journal of the National Cancer Institute*.

[6] Minghetti L (2005). Role of inflammation in neurodegenerative diseases. *Current Opinion in Neurology*.

[7] Wyss-Coray T, Mucke L (2002). Inflammation in neurodegenerative disease-a double-edged sword. *Neuron*.

[8] Krishnaswamy JK, Chu T, Eisenbarth SC (2013). Beyond pattern recognition: NOD-like receptors in dendritic cells. *Trends in Immunology*.

[9] Takeda K, Akira S (2004). TLR signaling pathways. *Seminars in Immunology*.

[10] O’Neill LA, Bowie AG (2007). The family of five: TIR-domain-containing adaptors in Toll-like receptor signalling. *Nature Reviews Immunology*.

[11] Medzhitov R (2001). Toll-like receptors and innate immunity. *Nature Reviews Immunology*.

[12] Mogensen TH (2009). Pathogen recognition and inflammatory signaling in innate immune defenses. *Clinical Microbiology Reviews*.

[13] Becker C, O’Neill LAJ (2007). Inflammasomes in inflammatory disorders: the role of TLRs and their interactions with NLRs. *Seminars in Immunopathology*.

[14] Tak PP, Firestein GS (2001). NF-*κ*B: a key role in inflammatory diseases. *Journal of Clinical Investigation*.

[15] Kelly RJ, Minogue AM, Lyons A (2013). Glial activation in AbetaPP/PS1 mice is associated with infiltration of IFNgamma-producing cells. *Journal of Alzheimer's Disease*.

[16] Takeuchi O, Akira S (2010). Pattern recognition receptors and inflammation. *Cell*.

[17] Proell M, Riedl SJ, Fritz JH, Rojas AM, Schwarzenbacher R (2008). The Nod-Like Receptor (NLR) family: a tale of similarities and differences. *PLoS ONE*.

[18] Shaw PJ, Lamkanfi M, Kanneganti T (2010). NOD-like receptor (NLR) signaling beyond the inflammasome. *European Journal of Immunology*.

[19] Kufer TA (2008). Signal transduction pathways used by NLR-type innate immune receptors. *Molecular BioSystems*.

[20] Duewell P, Kono H, Rayner KJ (2010). NLRP3 inflammasomes are required for atherogenesis and activated by cholesterol crystals. *Nature*.

[21] Eisenbarth SC, Colegio OR, O’Connor W, Sutterwala FS, Flavell RA (2008). Crucial role for the Nalp3 inflammasome in the immunostimulatory properties of aluminium adjuvants. *Nature*.

[22] Martinon F, Pétrilli V, Mayor A, Tardivel A, Tschopp J (2006). Gout-associated uric acid crystals activate the NALP3 inflammasome. *Nature*.

[23] Masters SL, O’Neill LAJ (2011). Disease-associated amyloid and misfolded protein aggregates activate the inflammasome. *Trends in Molecular Medicine*.

[24] Griffin WS, Sheng JG, Royston MC (1998). Glial-neuronal interactions in Alzheimer’s disease: the potential role of a ’cytokine cycle’ in disease progression. *Brain Pathology*.

[25] Ogura H, Murakami M, Okuyama Y (2008). Interleukin-17 promotes autoimmunity by triggering a positive-feedback loop via interleukin-6 induction. *Immunity*.

[26] Hanada T, Yoshimura A (2002). Regulation of cytokine signaling and inflammation. *Cytokine and Growth Factor Reviews*.

[27] Zheng Z, White C, Lee J (2010). Altered microglial copper homeostasis in a mouse model of Alzheimer’s disease. *Journal of Neurochemistry*.

[28] Lee KH, Yun S, Nam KN, Gho YS, Lee EH (2007). Activation of microglial cells by ceruloplasmin. *Brain Research*.

[29] Becaria A, Lahiri DK, Bondy SC (2006). Aluminum and copper in drinking water enhance inflammatory or oxidative events specifically in the brain. *Journal of Neuroimmunology*.

[30] Lu J, Wu D, Zheng Y (2009). Trace amounts of copper exacerbate beta amyloid-induced neurotoxicity in the cholesterol-fed mice through TNF-mediated inflammatory pathway. *Brain, Behavior, and Immunity*.

[31] Rossi-George A, Guo C-J, Oakes BL, Gow AJ (2012). Copper modulates the phenotypic response of activated BV2 microglia through the release of nitric oxide. *Nitric Oxide*.

[32] Maynard CJ, Cappai R, Volitakis I (2002). Overexpression of Alzheimer’s disease amyloid-*β* opposes the age-dependent elevations of brain copper and iron. *Journal of Biological Chemistry*.

[33] Bellingham SA, Lahiri DK, Maloney B, La Fontaine S, Multhaup G, Camakaris J (2004). Copper depletion down-regulates expression of the Alzheimer’s disease amyloid-*β* precursor protein gene. *Journal of Biological Chemistry*.

[34] Bailey SL, Carpentier PA, McMahon EJ, Begolka WS, Miller SD (2006). Innate and adaptive immune responses of the central nervous system. *Critical Reviews Immunology*.

[35] Bajramovic JJ (2011). Regulation of innate immune responses in the central nervous system. *CNS & Neurological Disorders Drug Targets*.

[36] Weller RO, Djuanda E, Yow H, Carare RO (2009). Lymphatic drainage of the brain and the pathophysiology of neurological disease. *Acta Neuropathologica*.

[37] Xiao B-G, Link H (1998). Immune regulation within the central nervous system. *Journal of the Neurological Sciences*.

[38] Kivisäkk P, Mahad DJ, Callahan MK (2003). Human cerebrospinal fluid central memory CD4+ T cells: evidence for trafficking through choroid plexus and meninges via P-selectin. *Proceedings of the National Academy of Sciences of the United States of America*.

[39] Kida S, Weller RO, Zhang E-T, Phillips MJ, Iannotti F (1995). Anatomical pathways for lymphatic drainage of the brain and their pathological significance. *Neuropathology and Applied Neurobiology*.

[40] Glass CK, Saijo K, Winner B, Marchetto MC, Gage FH (2010). Mechanisms underlying inflammation in neurodegeneration. *Cell*.

[41] Streit WJ, Mrak RE, Griffin WST (2004). Microglia and neuroinflammation: a pathological perspective. *Journal of Neuroinflammation*.

[42] Alexianu ME, Kozovska M, Appel SH (2001). Immune reactivity in a mouse model of familial ALS correlates with disease progression. *Neurology*.

[43] Autti T, Raininko R, Santavuori P, Vanhanen SL, Poutanen VP, Haltia M (1997). MRI of neuronal ceroid lipofuscinosis. II. Postmortem MRI and histopathological study of the brain in 16 cases of neuronal ceroid lipofuscinosis of juvenile or late infantile type. *Neuroradiology*.

[44] Bamberger ME, Harris ME, McDonald DR, Husemann J, Landreth GE (2003). A cell surface receptor complex for fibrillar *β*-amyloid mediates microglial activation. *Journal of Neuroscience*.

[45] Damier P, Hirsch EC, Zhang P, Agid Y, Javoy-Agid F (1993). Glutathione peroxidase, glial cells and Parkinson’s disease. *Neuroscience*.

[46] Ingelsson M, Fukumoto H, Newell KL (2004). Early A*β* accumulation and progressive synaptic loss, gliosis, and tangle formation in AD brain. *Neurology*.

[47] Kamo H, Haebara H, Akiguchi I (1987). A distinctive distribution of reactive astroglia in the precentral cortex in amyotrophic lateral sclerosis. *Acta Neuropathologica*.

[48] Thellung S, Florio T, Corsaro A (2000). Intracellular mechanisms mediating the neuronal death and astrogliosis induced by the prion protein fragment 106-126. *International Journal of Developmental Neuroscience*.

[49] Dickson DW, Farlo J, Davies P, Crystal H, Fuld P, Yen S-HC (1988). Alzheimer’s disease. A double-labeling immunohistochemical study of senile plaques. *American Journal of Pathology*.

[50] McGeer PL, Akiyama H, Itagaki S, McGeer EG (1989). Immune system response in Alzheimer’s disease. *Canadian Journal of Neurological Sciences*.

[51] Rozemuller JM, Eikelenboom P, Pals ST, Stam FC (1989). Microglial cells around amyloid plaques in Alzheimer’s disease express leucocyte adhesion molecules of the LFA-1 family. *Neuroscience Letters*.

[52] Jomova K, Vondrakova D, Lawson M, Valko M (2010). Metals, oxidative stress and neurodegenerative disorders. *Molecular and Cellular Biochemistry*.

[53] Valko M, Morris H, Cronin MTD (2005). Metals, toxicity and oxidative stress. *Current Medicinal Chemistry*.

[54] Akama KT, Albanese C, Pestell RG, Van Eldik LJ (1998). Amyloid *β*-peptide stimulates nitric oxide production in astrocytes through an NF*κ*b-dependent mechanism. *Proceedings of the National Academy of Sciences of the United States of America*.

[55] Flanders KC, Lippa CF, Smith TW, Pollen DA, Sporn MB (1995). Altered expression of transforming growth factor-*β* in Alzheimer’s disease. *Neurology*.

[56] Grammas P, Ovase R (2001). Inflammatory factors are elevated in brain microvessels in Alzheimer’s disease. *Neurobiology of Aging*.

[57] Lue LF, Rydel R, Brigham EF (2001). Inflammatory repertoire of Alzheimer’s disease and nondemented elderly microglia in vitro. *Glia*.

[58] Akiyama H, Streit W, Strohmeyer R (2000). Inflammation and Alzheimer's disease. *Neurobiology of Aging*.

[59] Salminen A, Ojala J, Kauppinen A, Kaarniranta K, Suuronen T (2009). Inflammation in Alzheimer’s disease: amyloid-*β* oligomers trigger innate immunity defence via pattern recognition receptors. *Progress in Neurobiology*.

[60] Mocchegiani E, Costarelli L, Giacconi R, Piacenza F, Basso A, Malavolta M (2012). Micronutrient (Zn, Cu, Fe)-gene interactions in ageing and inflammatory age-related diseases: implications for treatments. *Ageing Research Reviews*.

[61] Madaric A, Ginter E, Kadrabova J (1994). Serum copper, zinc and copper/zinc ratio in males: influence of aging. *Physiological Research*.

[62] Lawson LJ, Perry VH, Dri P, Gordon S (1990). Heterogeneity in the distribution and morphology of microglia in the normal adult mouse brain. *Neuroscience*.

[63] Mittelbronn M, Dietz K, Schluesener HJ, Meyermann R (2001). Local distribution of microglia in the normal adult human central nervous system differs by up to one order of magnitude. *Acta Neuropathologica*.

[64] Olah M, Amor S, Brouwer N (2012). Identification of a microglia phenotype supportive of remyelination. *Glia*.

[65] DeWitt DA, Perry G, Cohen M, Doller C, Silver J (1998). Astrocytes regulate microglial phagocytosis of senile plaque cores of Alzheimer’s disease. *Experimental Neurology*.

[66] Shaffer LM, Dority MD, Gupta-Bansal R, Frederickson RCA, Younkin SG, Brunden KR (1995). Amyloid *β* precursor protein (A*β*) removal by neuroglial cells in culture. *Neurobiology of Aging*.

[67] Meda L, Baron P, Prat E (1999). Proinflammatory profile of cytokine production by human monocytes and murine microglia stimulated with *β*-amyloid[25–35]. *Journal of Neuroimmunology*.

[68] Remarque EJ, Bollen ELEM, Weverling-Rijnsburger AWE, Laterveer JC, Blauw GJ, Westendorp RGJ (2001). Patients with Alzheimer’s disease display a pro-inflammatory phenotype. *Experimental Gerontology*.

[69] Colton CA, Gilbert DL (1987). Production of superoxide anions by a CNS macrophage, the microglia. *FEBS Letters*.

[70] Colton CA (2009). Heterogeneity of microglial activation in the innate immune response in the brain. *Journal of Neuroimmune Pharmacology*.

[71] Jimenez S, Baglietto-Vargas D, Caballero C (2008). Inflammatory response in the hippocampus of PS1M146L/APP751SL mouse model of Alzheimer’s disease: age-dependent switch in the microglial phenotype from alternative to classic. *Journal of Neuroscience*.

[72] Kimelberg H (1983). Primary astrocyte cultures—a key to astrocyte function. *Cellular and Molecular Neurobiology*.

[73] Farina C, Aloisi F, Meinl E (2007). Astrocytes are active players in cerebral innate immunity. *Trends in Immunology*.

[74] Mrak RE, Sheng JG, Griffin WST (1996). Correlation of astrocytic S100*β* expression with dystrophie neurites in amyloid plaques of Alzheimer’s disease. *Journal of Neuropathology and Experimental Neurology*.

[75] Pike CJ, Cummings BJ, Monzavi R, Cotman CW (1994). *β*-Amyloid-induced changes in cultured astrocytes parallel reactive astrocytosis associated with senile plaques in Alzeimer’s disease. *Neuroscience*.

[76] Wyss-Coray T, Loike JD, Brionne TC (2003). Adult mouse astrocytes degrade amyloid-*β* in vitro and in situ. *Nature Medicine*.

[77] Del Bo R, Angeretti N, Lucca E, De Simoni MG, Forloni G (1995). Reciprocal control of inflammatory cytokines, IL-1 and IL-6, *β*-amyloid production in cultures. *Neuroscience Letters*.

[78] Johnstone M, Gearing AJH, Miller KM (1999). A central role for astrocytes in the inflammatory response to *β*- amyloid; chemokines, cytokines and reactive oxygen species are produced. *Journal of Neuroimmunology*.

[79] Forloni G, Mangiarotti F, Angeretti N, Lucca E, De Simoni MG (1997). *β*-amyloid fragment potentiates IL-6 and TNF-*α* secretion by LPS in astrocytes but not in microglia. *Cytokine*.

[80] Hu J, Akama KT, Krafft GA, Chromy BA, Van Eldik LJ (1998). Amyloid-*β* peptide activates cultured astrocytes: morphological alterations, cytokine induction and nitric oxide release. *Brain Research*.

[81] Haass C, Selkoe DJ (2007). Soluble protein oligomers in neurodegeneration: lessons from the Alzheimer’s amyloid *β*-peptide. *Nature Reviews Molecular Cell Biology*.

[82] Walsh DM, Selkoe DJ (2007). A*β* oligomers-a decade of discovery. *Journal of Neurochemistry*.

[83] Harris ME, Hensley K, Butterfield DA, Leedle RA, Carney JM (1995). Direct evidence of oxidative injury produced by the Alzheimer’s *β*-amyloid peptide (1–40) in cultured hippocampal neurons. *Experimental Neurology*.

[84] Walter S, Letiembre M, Liu Y (2007). Role of the toll-like receptor 4 in neuroinflammation in Alzheimer’s disease. *Cellular Physiology and Biochemistry*.

[85] Jana M, Palencia CA, Pahan K (2008). Fibrillar amyloid-*β* peptides activate microglia via TLR2: implications for Alzheimer’s disease. *Journal of Immunology*.

[86] Chaney MO, Stine WB, Kokjohn TA (2005). RAGE and amyloid beta interactions: atomic force microscopy and molecular modeling. *Biochimica et Biophysica Acta*.

[87] Lue LF, Walker DG, Brachova L (2001). Involvement of microglial receptor for advanced glycation endproducts (RAGE) in Alzheimer’s disease: identification of a cellular activation mechanism. *Experimental Neurology*.

[88] Salminen A, Ojala J, Suuronen T, Kaarniranta K, Kauppinen A (2008). Amyloid-*β* oligomers set fire to inflammasomes and induce Alzheimer’s pathology: Alzheimer Review Series. *Journal of Cellular and Molecular Medicine*.

[89] Jack CS, Arbour N, Manusow J (2005). TLR signaling tailors innate immune responses in human microglia and astrocytes. *Journal of Immunology*.

[90] Kielian T (2006). Toll-like receptors in central nervous system glial inflammation and homeostasis. *Journal of Neuroscience Research*.

[91] Song M, Jin J, Lim J (2011). TLR4 mutation reduces microglial activation, increases A*β* deposits and exacerbates cognitive deficits in a mouse model of Alzheimer’s disease. *Journal of Neuroinflammation*.

[92] Richard KL, Filali M, Préfontaine P, Rivest S (2008). Toll-like receptor 2 acts as a natural innate immune receptor to clear amyloid *β*1-42 and delay the cognitive decline in a mouse model of Alzheimer’s disease. *Journal of Neuroscience*.

[93] Tahara K, Kim H, Jin J, Maxwell JA, Li L, Fukuchi K (2006). Role of toll-like receptor signalling in A*β* uptake and clearance. *Brain*.

[94] Stewart CR, Stuart LM, Wilkinson K (2010). CD36 ligands promote sterile inflammation through assembly of a Toll-like receptor 4 and 6 heterodimer. *Nature Immunology*.

[95] Zhang W, Wang L, Yu J, Chi Z, Tan L (2012). Increased expressions of TLR2 and TLR4 on peripheral blood mononuclear cells from patients with Alzheimer’s disease. *Journal of the Neurological Sciences*.

[96] Bierhaus A, Stern DM, Nawroth PP (2006). RAGE in inflammation: a new therapeutic target?. *Current Opinion in Investigational Drugs*.

[97] Deane R, Wu Z, Zlokovic BV (2004). RAGE (Yin) versus LRP (Yang) balance regulates Alzheimer amyloid *β*-peptide clearance through transport across the blood-brain barrier. *Stroke*.

[98] Donahue JE, Flaherty SL, Johanson CE (2006). RAGE, LRP-1, and amyloid-beta protein in Alzheimer’s disease. *Acta Neuropathologica*.

[99] Arancio O, Zhang HP, Chen X (2004). RAGE potentiates A*β*-induced perturbation of neuronal function in transgenic mice. *EMBO Journal*.

[100] Zuliani G, Ranzini M, Guerra G (2007). Plasma cytokines profile in older subjects with late onset Alzheimer’s disease or vascular dementia. *Journal of Psychiatric Research*.

[101] Ojala J, Alafuzoff I, Herukka S, van Groen T, Tanila H, Pirttilä T (2009). Expression of interleukin-18 is increased in the brains of Alzheimer’s disease patients. *Neurobiology of Aging*.

[102] Apelt J, Schliebs R (2001). *β*-amyloid-induced glial expression of both pro- and anti-inflammatory cytokines in cerebral cortex of aged transgenic Tg2576 mice with Alzheimer plaque pathology. *Brain Research*.

[103] Pétrilli V, Dostert C, Muruve DA, Tschopp J (2007). The inflammasome: a danger sensing complex triggering innate immunity. *Current Opinion in Immunology*.

[104] Furukawa K, Barger SW, Blalock EM, Mattson MP (1996). Activation of K+ channels and suppression of neuronal activity by secreted *β*-amyloid-precursor protein. *Nature*.

[105] Yu SP, Farhangrazi ZS, Ying HS, Yeh C, Choi DW (1998). Enhancement of outward potassium current may participate in *β*-amyloid peptide-induced cortical neuronal death. *Neurobiology of Disease*.

[106] Halle A, Hornung V, Petzold GC (2008). The NALP3 inflammasome is involved in the innate immune response to amyloid-*β*. *Nature Immunology*.

[107] Bush AI (2000). Metals and neuroscience. *Current Opinion in Chemical Biology*.

[108] Linder MC, Hazegh-Azam M (1996). Copper biochemistry and molecular biology. *American Journal of Clinical Nutrition*.

[109] Bayer TA, Schäfer S, Simons A (2003). Dietary Cu stabilizes brain superoxide dismutase 1 activity and reduces amyloid A*β* production in APP23 transgenic mice. *Proceedings of the National Academy of Sciences of the United States of America*.

[110] Lovell MA, Robertson JD, Teesdale WJ, Campbell JL, Markesbery WR (1998). Copper, iron and zinc in Alzheimer’s disease senile plaques. *Journal of the Neurological Sciences*.

[111] Schrag M, Crofton A, Zabel M (2011). Effect of cerebral amyloid angiopathy on brain iron, copper, and zinc in alzheimer’s disease. *Journal of Alzheimer’s Disease*.

[112] Noy D, Solomonov I, Sinkevich O, Arad T, Kjaer K, Sagi I (2008). Zinc-amyloid *β* interactions on a millisecond time-scale stabilize non-fibrillar Alzheimer-related species. *Journal of the American Chemical Society*.

[113] Smith MA, Harris PLR, Sayre LM, Perry G (1997). Iron accumulation in Alzheimer disease is a source of redox-generated free radicals. *Proceedings of the National Academy of Sciences of the United States of America*.

[114] Atwood CS, Moir RD, Huang X (1998). Dramatic aggregation of alzheimer by Cu(II) is induced by conditions representing physiological acidosis. *Journal of Biological Chemistry*.

[115] Atwood CS, Scarpa RC, Huang X (2000). Characterization of copper interactions with Alzheimer amyloid *β* peptides: identification of an attomolar-affinity copper binding site on amyloid *β*1–42. *Journal of Neurochemistry*.

[116] Curtain CC, Ali F, Volitakis I (2001). Alzheimer’s disease amyloid-*β* binds copper and zinc to generate an allosterically ordered membrane-penetrating structure containing superoxide dismutase-like subunits. *Journal of Biological Chemistry*.

[117] Huang X, Atwood CS, Moir RD, Hartshorn MA, Tanzi RE, Bush AI (2004). Trace metal contamination initiates the apparent auto-aggregation, amyloidosis, and oligomerization of Alzheimer’s A*β* peptides. *Journal of Biological Inorganic Chemistry*.

[118] Barnham KJ, Haeffner F, Ciccotosto GD (2004). Tyrosine gated electron transfer is key to the toxic mechanism of Alzheimer’s disease *β*-amyloid. *FASEB Journal*.

[119] White C, Lee J, Kambe T, Fritsche K, Petris MJ (2009). A role for the ATP7A copper-transporting ATPase in macrophage bactericidal activity. *Journal of Biological Chemistry*.

[120] Multhaup G, Schlicksupp A, Hesse L (1996). The amyloid precursor protein of Alzheimer’s disease in the reduction of copper(II) to copper(I). *Science*.

[121] Schmalz G, Schuster U, Schweikl H (1998). Influence of metals on IL-6 release in vitro. *Biomaterials*.

[122] Suska F, Esposito M, Gretzer C, Källtorp M, Tengvall P, Thomsen P (2003). IL-1*α*, IL-1*β* and TNF-*α* secretion during in vivo/ex vivo cellular interactions with titanium and copper. *Biomaterials*.

[123] Suska F, Gretzer C, Esposito M (2005). In vivo cytokine secretion and NF-*κ*B activation around titanium and copper implants. *Biomaterials*.

[124] Kennedy TP, Ghio AJ, Reed W (1998). Copper-dependent inflammation and nuclear factor-*κ*b activation by particulate air pollution. *American Journal of Respiratory Cell and Molecular Biology*.

[125] Rice TM, Clarke RW, Godleski JJ (2001). Differential ability of transition metals to induce pulmonary inflammation. *Toxicology and Applied Pharmacology*.

[126] Bar-Or D, Thomas GW, Yukl RL (2003). Copper stimulates the synthesis and release of interleukin-8 in human endothelial cells: a possible early role in systemic inflammatory responses. *Shock*.

[127] Hung YH, Bush AI, La Fontaine S (2013). Links between copper and cholesterol in Alzheimer's disease. *Frontiers in Physiology*.

[128] Terwel D, Löschmann Y, Schmidt HH-J, Schöler HR, Cantz T, Heneka MT (2011). Neuroinflammatory and behavioural changes in the Atp7B mutant mouse model of Wilson’s disease. *Journal of Neurochemistry*.

[129] Liu HP, Lin WY, Wang WF (2013). Genetic variability in copper-transporting P-type adenosine triphosphatase (ATP7B) is associated with Alzheimer's disease in a Chinese population. *Journal of Biological Regulators and Homeostatic Agents*.

[130] Squitti R, Polimanti R (2012). Copper hypothesis in the missing hereditability of sporadic alzheimer’s disease: ATP7B gene as potential harbor of rare variants. *Journal of Alzheimer’s Disease*.

[131] Solerte SB, Cravello L, Ferrari E, Fioravanti M (2000). Overproduction of IFN-*γ* and TNF-*α* from natural killer (NK) cells is associated with abnormal NK reactivity and cognitive derangement in Alzheimer’s disease. *Annals of the New York Academy of Sciences*.

[132] Baron R, Nemirovsky A, Harpaz I, Cohen H, Owens T, Monsonego A (2008). IFN-*γ* enhances neurogenesis in wild-type mice and in a mouse model of Alzheimer’s disease. *FASEB Journal*.

[133] Mastrangelo MA, Sudol KL, Narrow WC, Bowers WJ (2009). Interferon-*γ* differentially affects Alzheimer’s disease pathologies and induces neurogenesis in triple transgenic-AD mice. *American Journal of Pathology*.

[134] Connor JR, Tucker P, Johnson M, Snyder B (1993). Ceruloplasmin levels in the human superior temporal gyrus in aging and Alzheimer’s disease. *Neuroscience Letters*.

[135] Loeffler DA, LeWitt PA, Juneau PL (1996). Increased regional brain concentrations of ceruloplasmin in neurodegenerative disorders. *Brain Research*.

[136] Osaki S, Johnson DA (1969). Mobilization of liver iron by ferroxidase (ceruloplasmin). *Journal of Biological Chemistry*.

[137] Nemeth E, Tuttle MS, Powelson J (2004). Hepcidin regulates cellular iron efflux by binding to ferroportin and inducing its internalization. *Science*.

[138] Venti A, Giordano T, Eder P (2004). Integrated role of desferrioxamine and phenserine targeted to an iron-responsive element in the APP-mRNA 5′-untranslated region. *Annals of the New York Academy of Sciences*.

[139] Myhre O, Utkilen H, Duale N, Brunborg G, Hofer T (2013). Metal dyshomeostasis and inflammation in Alzheimer's and Parkinson's diseases: possible impact of environmental exposures. *Oxidative Medicine and Cellular Longevity*.

[140] Acevedo KM, Hung YH, Dalziel AH (2011). Copper promotes the trafficking of the amyloid precursor protein. *Journal of Biological Chemistry*.

[141] Zucconi GG, Cipriani S, Scattoni R, Balgkouranidou I, Hawkins DP, Ragnarsdottir KV (2007). Copper deficiency elicits glial and neuronal response typical of neurodegenerative disorders. *Neuropathology and Applied Neurobiology*.

[142] Liang WS, Dunckley T, Beach TG (2007). Gene expression profiles in anatomically and functionally distinct regions of the normal aged human brain. *Physiological Genomics*.

[143] Liang WS, Reiman EM, Valla J (2008). Alzheimer's disease is associated with reduced expression of energy metabolism genes in posterior cingulate neurons. *Proceedings of the National Academy of Sciences of the United States of America*.

[144] Agullo L, Garcia A, Hidalgo J (1998). Metallothionein-I+II induction by zinc and copper in primary cultures of rat microglia. *Neurochemistry International*.

[145] Crouch PJ, Hung LW, Adlard PA (2009). Increasing Cu bioavailability inhibits A*β* oligomers and tau phosphorylation. *Proceedings of the National Academy of Sciences*.

